# The association between genetically proxied GLP-1 receptor agonists and cancer risks

**DOI:** 10.1097/MS9.0000000000005253

**Published:** 2026-06-16

**Authors:** Fei Zhou, Zhao Yang, Maoying Xing, Geng Zong, Chengwu Feng, Jing Wang, Xin Geng

**Affiliations:** a Department of Medical Oncology, Cancer Center, Shanghai General Hospital, Shanghai Jiao Tong University School of Medicine, Shanghai, China; b Shanghai Eastern Hepatobiliary Surgery Hospital, Naval Medical University, Shanghai, China; c National Center for Liver Cancer, Naval Medical University, Shanghai, China; d Institute of Nutrition, Fudan University, Shanghai, China; e CAS Key Laboratory of Nutrition, Metabolism and Food Safety, Shanghai Institute of Nutrition and Health, University of Chinese Academy of Sciences, Chinese Academy of Sciences, Shanghai, China; f Department of Respiratory Medicine, Jinshan Hospital, Fudan University, Shanghai, P. R. China

**Keywords:** cancer, cohort study, diabetes, drug-target Mendelian randomization, GLP-1 receptor agonists, weight loss

## Abstract

Glucagon-like peptide-1 receptor agonists (GLP-1RAs) are widely used for type 2 diabetes and obesity, but their long-term cancer risks remain controversial in observational studies. We conducted a drug-target Mendelian randomization (MR) analysis using genetic variants in the GLP1R gene region as instruments to proxy GLP-1RA exposure. Summary-level data for 21 cancer types were obtained from public GWAS consortia. Inverse-variance weighted (IVW) MR was used as the primary method, supplemented by sensitivity analyses, Cox proportional hazards regression analyses, and mediation testing for BMI and HbA1c. Genetically proxied GLP-1RA use was associated with a reduced risk of breast cancer [the log of the odds ratio (log{OR}) = −0.343, *P* = 6.46 × 10^−4^] and prostate cancer [log(OR) = −0.004, *P* = 2.25 × 10^−4^], but an increased risk of head and neck cancer [log(OR) = 0.350, *P* = 1.17 × 10^−4^] and thyroid cancer [log(OR) = 1.016, *P* = 9.12 × 10^−4^]. The directions of these effects were validated by the Cox proportional hazards regression model using UK Biobank data and weighted polygenic scores for GLP1R expression, though the association did not reach significance (*P* > 0.05). Mediation analyses indicated that BMI explained about 3% of the causal effects on endometrial cancer, and HbA1c level explained 3% on head and neck cancer and 4% on thyroid cancer. This MR study provides evidence that GLP-1RA use may causally influence the risk of several cancers. These findings support the need for cancer-specific monitoring in patients using GLP-1RAs and further mechanistic research.

## Introduction

Glucagon-like peptide-1 receptor agonists (GLP-1RAs), first introduced for type 2 diabetes (T2D), have gained prominence as groundbreaking treatments for obesity, offering dual benefits in weight management and cardiometabolic risk reduction^[^1^]^. Yet, their expanding use, particularly at higher doses for obesity, has raised concerns regarding potential oncological consequences.

HIGHLIGHTS

- Novel Genetic Instrument: This study employs a robust drug-target Mendelian randomization (MR) design, utilizing genetic variants in the *GLP1R* gene region as proxies for GLP-1 receptor agonist (GLP-1RA) exposure to infer causal effects on cancer risk.
- Complex Cancer Risk Profile: Genetically proxied GLP-1RA use was causally associated with a reduced risk of breast and prostate cancers, but an increased risk of head and neck cancer, as well as thyroid cancer.
- Multi-Method Validation: The primary MR findings were consistently supported by the replication cohort and sensitivity analyses, and the direction of effect was validated in an independent cohort from the UK Biobank using polygenic risk scores.
- Mediation by Metabolic Traits: Mediation analyses revealed that the causal effects on specific cancers were minimally explained (≤5%) by changes in BMI or HbA1c levels, suggesting the involvement of other biological pathways.

Current epidemiological and clinical data reveal a complex risk profile, and observational studies are inherently confounded by immortal time bias, survival bias, and heterogeneous exposure definitions (e.g., off-label obesity use vs. T2D treatment). Randomized controlled trials, though less prone to bias, often lack sufficient duration or power to assess cancer outcomes. Temporal inadequacy plagues most trials with <5-year follow-up, which is insufficient for solid tumor latency. Confounding by indication persists where metabolic status influences prescribing, as sicker patients often receive insulin, elevating comparator group risk and potentially amplifying perceived GLP-1RA benefits^[^2^]^. Given these inconsistencies and limitations, advanced approaches, such as drug-target Mendelian randomization (MR) to minimize confounding and mechanistic studies of GLP-1R signaling in tumorigenesis, are critical to elucidate causal relationships.

MR leverages genetic variants as instrumental variables (IVs) to infer causal relationships between modifiable exposures and disease outcomes. This approach exploits the random assortment of alleles during meiosis, which mimics randomization in clinical trials^[^3^]^. Crucially, MR overcomes fundamental limitations of observational studies, including confounding by lifestyle factors and selection bias, by utilizing germline genetic variants fixed at conception^[^3^]^. The methodology relies on three core assumptions: (1) robust association between instruments and exposure (relevance), (2) no association with confounders (independence), and (3) exclusive effect on outcomes through the exposure (exclusion restriction). Sensitivity analyses address violations, such as horizontal pleiotropy, through MR-Egger regression and MR-PRESSO. Drug-target MR could employ genetic variants in or near drug target genes to mimic the pharmacological effect of drug-target genes^[^4^]^. Moreover, two-step MR mediation analysis could help explore the mechanism underlying the causal relationship.

Although the causality between GLP-1RA and cancers was examined by MR^[^5^]^, a critical methodological flaw in the selection of IVs might undermine the reliability of its findings^[^6^]^. This study aims to address existing gaps by integrating genetic and longitudinal clinical data to evaluate the cancer safety profile of GLP-1RAs. In addition, we aim to resolve whether causal effects are primarily driven by BMI or fasting glucose level mediation, informing targeted cancer prevention strategies.

## Methods

### Study design for MR

**Figure 1.**
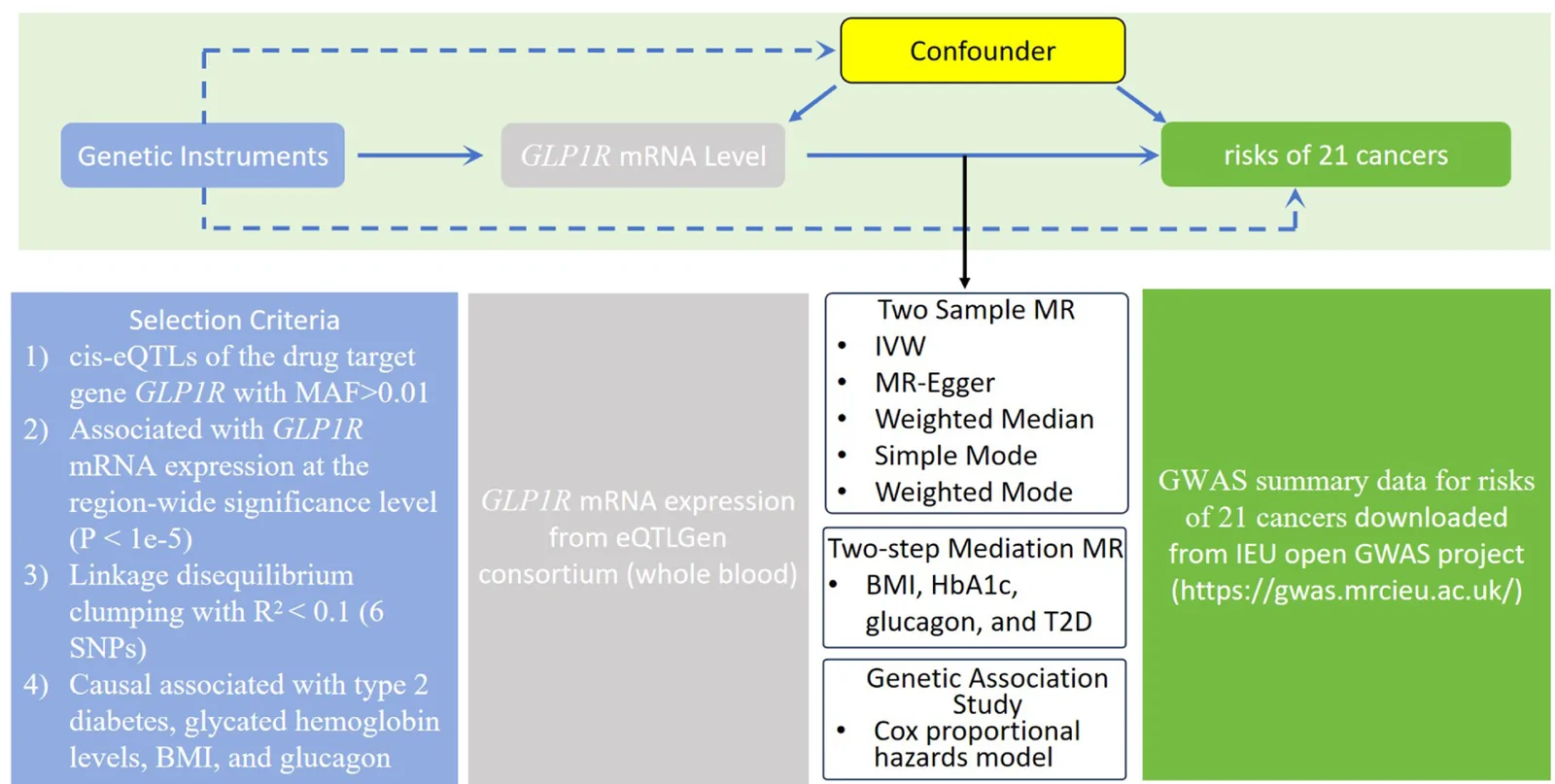
**Study design.** Two-sample Mendelian randomization (MR) analyses of the effect of GLP-1RA on 21 cancers were conducted. Summary data of exposure and outcomes were obtained from related genome-wide association analyses. The inverse variance-weighted (IVW) approach was applied as the primary method to estimate the causal effect on selected outcomes. Several sensitivity analyses were conducted. Genetic association analyses using the UK Biobank individual-level data were conducted to validate our results. We also conducted two-step mediation MR. GLP-1R, glucagon-like peptide-1 receptor; IVW, inverse variance weighted; LD, linkage disequilibrium; SNP, single-nucleotide polymorphism.

In line with the STROCSS guidelines^[^7^]^, this investigation adopted a two-sample drug-target MR framework to examine putative causal effects of GLP-1RAs on cancer risk. The analytical strategy, schematically represented in Figure 1, encompassed three core components: selection of genetic instruments proxying GLP-1RA pharmacological action, curation of cancer-related outcome datasets, and application of robust MR analytical techniques. Primary causal estimates were derived using the inverse variance-weighted (IVW) method, with sensitivity analyses performed via MR-Egger, weighted median, simple mode, and weighted mode estimators to evaluate result consistency and pleiotropic influences.

### Selection and validation of genetic predictors for GLP-1RAs

Genetic proxies for sustained GLP-1RA exposure were selected through a multi-tiered process. Cis-expression quantitative trait loci (cis-eQTLs) associated with GLP1R were initially identified within whole blood transcriptomic data from the eQTLGen Consortium^[^8^]^. Variants meeting criteria for common frequency (minor allele frequency > 1%) and exhibiting strong statistical significance (*P* < 1 × 10^−5^) with GLP1R expression were retained. Linkage disequilibrium (LD) clumping (*R*^2^ < 0.1) yielded six independent SNPs. Instrument validity was further corroborated through positive control analyses assessing associations with type 2 diabetes and HbA1c levels. The strength of genetic instruments was quantified via F-statistics, with all values surpassing the conventional threshold of 10, indicating minimal weak instrument bias.

### Outcome sources and replications for MR

Summary-level genetic association data for 21 malignancy phenotypes were sourced from the IEU GWAS database (https://gwas.mrcieu.ac.uk/), including colorectal cancer, breast cancer, thyroid cancer, endometrial cancer, ovarian cancer, head and neck cancer, prostate cancer, basal cell carcinoma, melanoma, bladder cancer, lung cancer, cervical cancer, pancreatic cancer, gastric cancer, liver cancer, esophageal cancer, kidney cancer, malignant lymphoma, leukemia, multiple myeloma, and brain cancer. When available, estimates were corroborated using independent cohort resources.

### Statistical analyses for MR

Analyses were conducted utilizing the “TwoSampleMR” package (v0.6.15) in R. Causal estimates for the relationship between GLP1R expression-associated variants and cancer outcomes were primarily computed via the IVW approach. Potential pleiotropy was interrogated using the MR-Egger intercept test, and heterogeneity across variants was assessed via Cochran’s Q statistic. A Bonferroni-adjusted significance threshold (*P* < 0.002, accounting for 21 outcomes) was applied for primary analyses, with *P* < 0.05 considered suggestive of association. Mediation analyses employing the “RMediation” package were implemented to explore potential intermediary roles of glucagon, BMI, T2D, and HbA1c.

### Population of the observational genetic association study

The UK Biobank (UKB) is a prospective cohort comprising over 500 000 individuals aged 40–69 years, recruited from 22 assessment centers across the UK between 2006 and 2010^[^9^]^. The cohort integrates large-scale genomic data with deep phenotypic characterization, including physical measures, biological samples, and linked electronic health records. All participants provided electronic informed consent, and ethical approval was obtained from relevant UK national committees.

The analysis excluded participants who withdrew consent (*n* = 569), had missing genetic data (*n* = 15,190), were of non-European ancestry (*n* = 78,269), exhibited sex discrepancies (*n* = 301), showed excess relatedness (*n* = 161), had putative aneuploidy (*n* = 407), had a cancer diagnosis (*n* = 40,270), or had diabetes (*n* = 19,699) at baseline. To account for sex-specific cancers, the analysis was restricted to males for prostate cancer and to females for endometrial and ovarian cancers. The final analytical sample size for each cancer site is presented in Supplemental Digital Content Figure S1, available at: http://links.lww.com/MS9/B250.

### Weighted polygenic risk score

Genotyping in UKB was performed using the UK BiLEVE and UKB Axiom arrays, with imputation carried out against the UK10K and 1000 Genomes Phase 3 reference panels^[^9^]^. The polygenic scores (PGS) for GLP1R expression were constructed by aggregating six cis-eQTL SNPs (imputation quality score > 0.8), each weighted by its respective effect on GLP1R expression. Participants were stratified into tertiles based on the PGS distribution.

### Covariates for observational genetic association study

Covariate data were collected at baseline via standardized questionnaires, interviews, and physical examinations. These included age, sex, education, household income, smoking behavior, alcohol consumption, and physical activity levels. The Townsend deprivation index, an area-level socioeconomic metric, was assigned based on postal code. A healthy diet was defined as meeting adequate intake recommendations for four or more of seven key food groups associated with cardiometabolic health, including sufficient consumption of fruits, vegetables, whole grains, fish, and vegetable oils, along with limited intake of refined grains and sugar-sweetened beverages^[^10^]^. Participants were classified as meeting physical activity guidelines if they reported ≥ 150 minutes of moderate or ≥ 75 minutes of vigorous activity weekly^[^11^]^. Anthropometric measurements were obtained by trained staff following a standard protocol at enrollment. Body mass index was computed as weight in kilograms divided by the square of height in meters.

### Outcome ascertainment

We included UKB participants who were free of cancer at baseline. Incident cancer cases were identified through linkages to hospital inpatient records, national death registries, national cancer registries, and self-reported data (for details, see Supplemental Digital Content Table S1, available at: http://links.lww.com/MS9/B250). Person-time of follow-up was calculated from the date of baseline assessment until the date of the first occurrence of cancer, death, or the administrative censoring date, whichever came first. The administrative censoring dates were 31 December 2020 for participants in England, 31 December 2016 for those in Wales, and 30 November 2021 for those in Scotland.

### Statistical analysis for observational study

Missing data were handled via median/mode imputation for variables with ≤5% missingness; otherwise, a missing indicator category was created. Associations between GLP1R PGS and cancer incidence were evaluated using Cox proportional hazards models, with person-time calculated from enrollment until diagnosis, death, or censoring. Proportional hazards assumptions were verified using Schoenfeld residuals. Multivariable models were adjusted for age, sex, education, Townsend index, smoking, alcohol use, physical activity, diet, BMI, and genetic principal components. Trend tests were performed by modeling the median PGS value per tertile as a continuous variable. All analyses were conducted in R v4.4.0.

## Results

A total of six independent cis-eQTL SNPs were selected as genetic instruments for the drug target gene GLP1R (Supplemental Digital Content Table S2, available at: http://links.lww.com/MS9/B250). All IVs exhibited F statistics greater than 10, indicating minimal risk of weak instrument bias. Furthermore, positive control analyses confirmed that the IVs were significantly associated with a reduced risk of T2D, as well as lower levels of HbA1c, glucagon, and BMI (*P* < 0.05; Supplemental Digital Content Table S3, available at: http://links.lww.com/MS9/B250). No significant heterogeneity or horizontal pleiotropy was detected, further supporting the validity of the selected genetic instruments.

**Figure 2.**
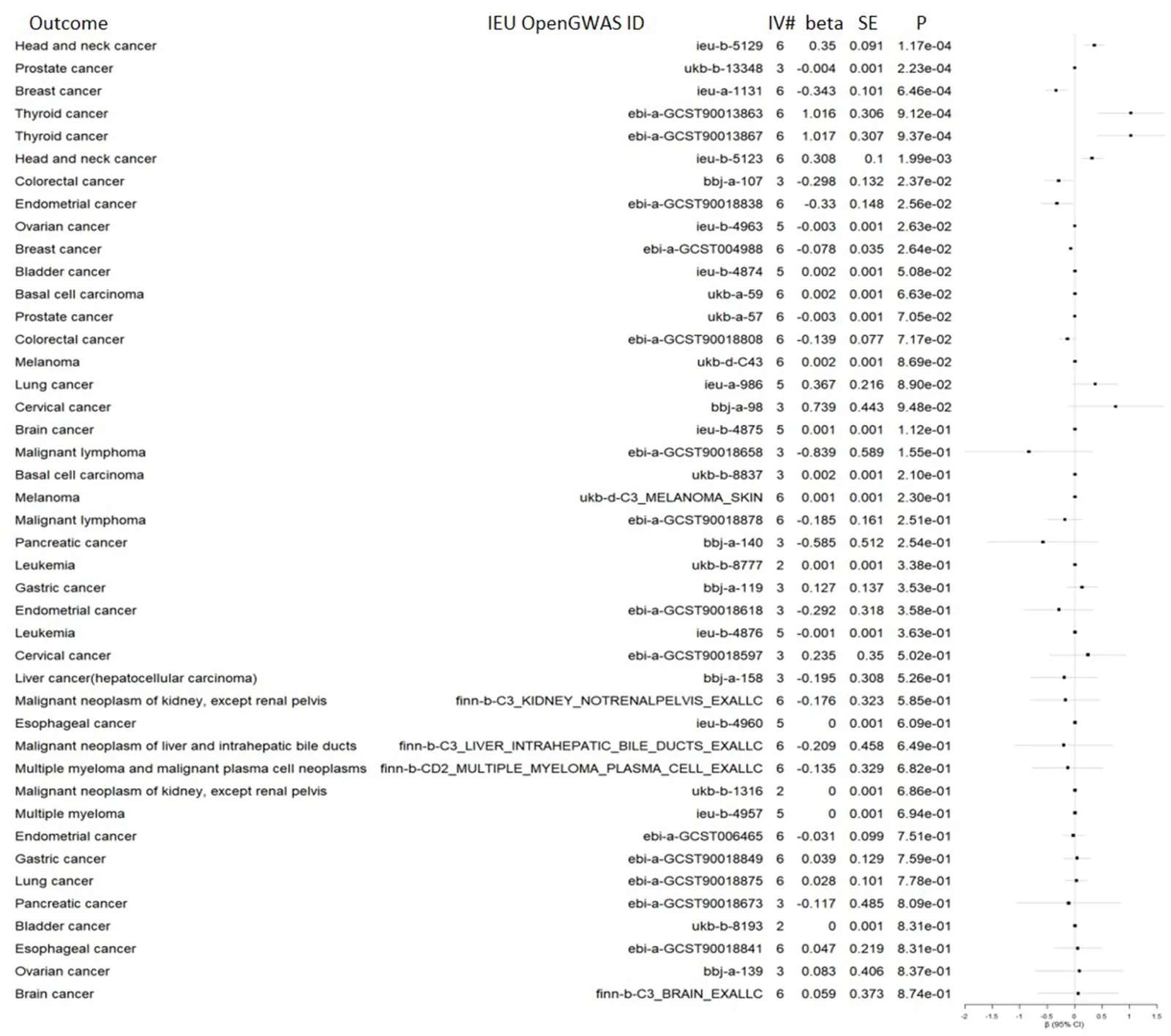
Causal effect of genetically proxied glucagon-like peptide-1 receptor agonists on cancer outcomes by inverse variance-weighted model.

Based on IVW MR results, GLP-1RA was significantly associated with an increased risk of head and neck cancer (IEU OpenGWAS ID: ieu-b-5129; log(OR) = 0.350; *P* = 1.17 × 10^−4^) (Fig. 2). This association was corroborated by sensitivity analyses using the weighted median method (*P* = 4.07 × 10^−3^) and simple mode (*P* = 0.038), and was further replicated in an independent cohort (ID: ieu-b-5123; IVW *P* = 1.99 × 10^−3^; simple mode *P* = 0.038).

A significant decrease in breast cancer risk was associated with GLP-1RA [ID: ieu-a-1131; log(OR) = −0.343; IVW *P* = 6.46 × 10^−4^], which was supported by both weighted median (*P* = 1.22 × 10^−3^) and weighted mode (*P* = 0.038) methods. This association was nominally significant in another cohort (ID: ebi-a-GCST004988; IVW *P* = 0.026).

GLP-1RA was significantly associated with an increased risk of thyroid cancer in two independent cohorts [ID: ebi-a-GCST90013863; log(OR) = 1.016; IVW *P* = 9.12 × 10^−4^; ID: ebi-a-GCST90013867; log(OR) = 1.017; IVW *P* = 9.36 × 10^−4^], with validation via the weighted median method.

A significant reduction in prostate cancer risk was observed in relation to GLP-1RA [ID: ukb-b-13348; log(OR) = −0.004; IVW *P* = 2.25 × 10^−4^], which was confirmed by the weighted median method (*P* = 3.21 × 10^−3^). Significance (*P* < 0.05) was also observed in an additional cohort (ID: ukb-a-57) using three alternative methods, though not with IVW.

Additionally, nominally significant reductions in the risks of colorectal, endometrial, and ovarian cancer were associated with GLP-1RA via IVW (*P* < 0.05). However, these associations were identified in only one cohort and were not validated in sensitivity analyses. Moreover, a nominally increased risk of basal cell carcinoma was associated with GLP-1RA only in the weighted median analysis within the UKB-a-59 cohort (*P* < 0.05; Supplemental Digital Content Table S4, available at: http://links.lww.com/MS9/B250). Although no significant association was found for lung cancer risk, the risk of squamous cell lung cancer was nominally associated with GLP-1RA (ID: IEU-a-967; *P* < 0.05; Supplemental Digital Content Table S4, available at: http://links.lww.com/MS9/B250).

The heterogeneity and horizontal pleiotropy for all of these suggestive and significant associations were not significant. Associations between GLP1R PGS and cancer incidence using UKB data, analyzed by Cox proportional hazards models, validated the directions of the results from MR analyses, although all the *P* > 0.05 (Table 1).

**Table 1:** Associations between polygenic scores of GLP-1RAs and cancer risk in UK Biobank.

			Hazard ratios			
Cancer subtypes	No. of cases	Person-years	T1	T2	T3	*P* for trend
Head and neck	1088	4 111 032	1.00 (reference)	1.06 (0.91, 1.22)	1.04 (0.90, 1.19)	0.59
Prostate	8966	1 837 719	1.00 (reference)	0.94 (0.89, 0.99)	0.98 (0.93, 1.02)	0.26
Breast	7879	4 069 920	1.00 (reference)	0.92 (0.88, 0.98)	0.99 (0.94, 1.04)	0.45
Thyroid	336	4 114 971	1.00 (reference)	1.15 (0.89, 1.49)	1.04 (0.81, 1.35)	0.69
Colorectal	4727	4 090 743	1.00 (reference)	0.99 (0.92, 1.06)	0.95 (0.89, 1.02)	0.18
Endometrium	1195	2 224 870	1.00 (reference)	0.98 (0.85, 1.13)	0.97 (0.85, 1.11)	0.69
Ovary	1018	2 225 801	1.00 (reference)	0.93 (0.79, 1.08)	0.97 (0.84, 1.12)	0.65

Hazard ratios and 95% confidence intervals were calculated using Cox proportional hazards regression. The multivariable model was adjusted for the following covariates: demographic and socioeconomic factors [age, sex (where appropriate), educational attainment, Townsend deprivation index], lifestyle factors (smoking status, drinking status, physical activity, healthy diet, body mass index), and the first 10 genetic principal components. Trend tests were performed by modeling the median polygenic score value per tertile as a continuous variable. SD, standard deviation.

In two-step MR analyses, we evaluated the potential mediating roles of glucagon, BMI, T2D, and HbA1c levels in the association between GLP-1RA and cancer risks. Using IVW two-sample MR as the primary method, we detected significant causal effects of BMI on both endometrial cancer and squamous cell lung cancer, as well as of HbA1c levels on head and neck cancer, thyroid cancer, and squamous cell lung cancer (all *P* < 0.05; Supplemental Digital Content Table S5, available at: http://links.lww.com/MS9/B250). However, the proportion of the mediation effects attributable to these factors was small, all being close to or below 5%.

## Discussion

Our MR analysis revealed complex associations between GLP-1RA effects and cancer risks, including protective effects against breast and prostate cancers, but an elevated risk for head and neck cancer and thyroid cancer. Discrepancies may exist between MR findings and clinical trial or observational data.

Our finding of an increased thyroid cancer risk aligned with a 6-year nested case–control study from the French National Health Cancer Data System^[^12^]^. A retrospective cohort study by Wang et al. also suggested a potential link between GLP-1RAs and thyroid cancer, though not statistically significant^[^2^]^. A meta-analysis indicated that GLP-1RA treatment might be associated with a moderate increase in relative risk of thyroid cancer in clinical trials, alongside a slight rise in absolute risk^[^13^]^. Supporting these observations, a rodent study reported that continuous exposure to GLP-1R agonists led to elevated plasma calcitonin levels and a higher incidence of thyroid C-cell hyperplasia in wild-type mice^[^14^]^.

The reduced risk of colorectal cancer identified in our study is consistent with several retrospective cohort studies and meta-analyses^[^2,15,16^]^. Further supporting this, Zhu *et al* provided a comprehensive assessment of GLP-1 signaling-related genes, highlighting its protective role in colorectal cancer^[^17^]^. Contrary to our results indicating a decreased risk for breast cancer, some observational studies found no or increased risk of breast cancer associated with GLP-1RAs^[^2,18,19^]^.

Unlike other cancers discussed, there is limited prior evidence regarding the association of head and neck cancer risk with GLP-1RA. Oral cavity cancer is a common clinical manifestation of head and neck cancer, and squamous cell carcinoma is the predominant histological type. Thus, we specifically examined oral cavity cancer (ID: ieu-a-967, IVW *P* = 0.0349) and squamous cell carcinoma (ID: ukb-a-60, IVW *P* = 0.0456) using MR (Supplemental Digital Content Table S1, available at: http://links.lww.com/MS9/B250), which may explain the association between the risk of head and neck cancer and GLP-1RAs.

There are some limitations in our study. Blood eQTL-derived instruments may not accurately capture GLP1R expression in relevant tissues, such as thyroid C-cells. Moreover, MR assumes linear exposure–outcome relationships, which may not capture dose-dependent or threshold effects of GLP-1RAs seen in clinical settings. The limited genetic diversity in the underlying GWAS, with participants being of European ancestry, constrains the generalizability of the findings. Survival bias introduces further uncertainty, especially for cancers with long latency periods.

## Data Availability

All the data utilized in this study can be downloaded from the IEU Open GWAS Project and UKB.

## Research registration unique identifying number (UIN)

Research registration: not applicable, according to the law of China.
